# PeptideWitch–A Software Package to Produce High-Stringency Proteomics Data Visualizations from Label-Free Shotgun Proteomics Data

**DOI:** 10.3390/proteomes8030021

**Published:** 2020-08-21

**Authors:** David C. L. Handler, Flora Cheng, Abdulrahman M. Shathili, Paul A. Haynes

**Affiliations:** 1 Department of Molecular Sciences, Macquarie University, North Ryde, NSW 2109, Australia; david.handler@students.mq.edu.au (D.C.L.H.); abdulrahman-mansour-m.shath@hdr.mq.edu.au (A.M.S.); 2 Faculty of Medicine, Health and Human Sciences, Macquarie University, North Ryde, NSW 2109, Australia; flora.cheng@mq.edu.au

**Keywords:** label-free shotgun proteomics, false discovery rate, data quality, protein quantitation, spectral counting

## Abstract

PeptideWitch is a python-based web module that introduces several key graphical and technical improvements to the Scrappy software platform, which is designed for label-free quantitative shotgun proteomics analysis using normalised spectral abundance factors. The program inputs are low stringency protein identification lists output from peptide-to-spectrum matching search engines for ‘control’ and ‘treated’ samples. Through a combination of spectral count summation and inner joins, PeptideWitch processes low stringency data, and outputs high stringency data that are suitable for downstream quantitation. Data quality metrics are generated, and a series of statistical analyses and graphical representations are presented, aimed at defining and presenting the difference between the two sample proteomes.

## 1. Introduction

In the field of discovery proteomics, the aim of an experiment is to take a series of two or more biological samples and quantify the proteins within each. In its simplest version this experiment takes the form of a pairwise comparison. The desired output is the identification of all proteins in both samples as well as a classification of which proteins are more abundant in one sample or the other, and by how much.

One way to achieve this goal is the use of label-free quantitative shotgun proteomics methodologies. There are several different approaches available for label-free quantitation, including analysis of area under the curve (AUC) intensities for peptides and spectral counts (SpCs) [1]. Each method requires appropriate downstream analysis workflows to ensure the best quantitation takes place. Spectral counting has become a widely used option when analysing label-free data [2], and produces high-quality results, as demonstrated in a recent comprehensive comparative analysis between labelling with isobaric tags for relative and absolute quantification (iTRAQ), exponentially modified protein abundance index (emPAI), AUC and SpC identification workflows [3]. Since it does not involve expensive labelling reagents, the use of SpCs in proteomics workflows is a desirable option for researchers interested in holistic approaches to discovery proteomics.

Using spectral counts for protein identification and quantitation is not without drawbacks. The main weakness in using SpCs appears to be in clearly differentiating proteins with relatively small changes in expression, and for experimental samples with low abundance counts or small replicate numbers [1,4]. There are, however, several methods available which can help address some of the limitations of spectral counting for quantitation. One modified form of spectral counting which has become very widely used is the calculation of normalised spectral abundance factors (NSAFs) as measures of protein abundance, which takes into consideration the fact that larger proteins will produce more peptides given their length, and thus will have more chances to be measured in a spectrometer, and factors this into the identification process [5,6].

Our laboratory employs a modified workflow based on calculation of NSAF values for a minimum of three replicates for each biological sample. The approach aims to create a list of high-stringency protein identifications that improve quantitative biological veracity in subsequent downstream analysis. We achieve this by using NSAFs for quantitation as part of a process known as minimum spectral counting (MSC) [7,8]. MSC involves filtering low-stringency data outputs from a peptide-to-spectrum-matching (PSM) search engine, and collating protein identifiers that are present in all sample replicates of at least one sample, whilst ensuring that their raw spectral counts are summed to a user-specified minimum value (usually ≥ 5), prior to NSAF calculation. The reproducibly identified proteins collated as part of the MSC process represent a much higher stringency dataset since most of the random noise in the low-stringency data is filtered out because it is, by definition, not reproducible. The combination of MSC and NSAF is implemented in a series of R modules we created previously, known as Scrappy [7]. The implementation of high-stringency data rules in addition to NSAF production allows us to create a highly refined dataset that overcomes many of the weaknesses of using raw spectral counting. This is especially the case regarding the lability of low-fold-change proteins, where the criteria of minimum spectral counts summed across replicates remove much of the noise associated with identifying low abundance proteins. This analytical approach has been widely used in recent years, resulting in a diverse array of publications that span agricultural [9,10,11,12,13,14], parasitological [15,16,17,18] and medical [7,19,20,21] proteomics.

Here, we introduce a web module called PeptideWitch that produces high-stringency spectral counting data and conducts automated downstream quantitation. PeptideWitch not only produces high-stringency MSC and NSAF data from a range of PSM search engine outputs, it also expands greatly upon the functionality of the Scrappy R software [7] by producing additional statistical and graphical outputs for inter-and intra-replicate analysis, including Venn diagrams, volcano plots, heatmaps, and *p*-value histograms. PeptideWitch also incorporates a newly developed multiple testing corrections method [22] that utilises internal replicate permutation analysis of six replicates of a reference sample to produce an experimentally derived Benjamini-Hochberg (BH) corrected *p*-value threshold for subsequent application in control vs. treatment quantitation [23,24,25]. Lastly, PeptideWitch is freely web accessible so it can easily be used in any laboratory, since it does not require expertise in R (or Python) programming and implementation. We outline in the Methods the design of PeptideWitch, and present in the Results outputs from an example dataset that has been analysed using the platform.

## 2. Methods

### 2.1. Software Construction

The PeptideWitch software is currently at version 3.0 and was written in Python and flask, with pipenv also required for compilation. The code versioning system employed was git, and the legal code licence applicable is MIT. The permanent link to a code repository, including developer documentation, is available at [26].

### 2.2. Software Description

An up-to-date and web-accessible version of PeptideWitch can be found at: http://peptidewitch.online. For PeptideWitch analysis, users begin with standard output csv files from PSM search engines including X!Tandem running under Global Proteome Machine (GPM) [27], MetaMorpheus [28], or Proteome Discoverer [29]. Example data files can be found on peptidewitch.online; input files should contain lists of protein identifications alongside spectral counts, scores and protein molecular weights. Care must be taken with filenames and must conform to the following template: {state}-{R#}.csv, where R# refers to the replicate number. Files are uploaded to the server and processed, with a zip file output being returned to the user. Inputting three csv files representing replicates of a control set of data, and three replicate csv files representing a ‘treatment’ set of data, will yield classes of results as described in the following subsections, divided into separate subfolders.

**Figure 1:**
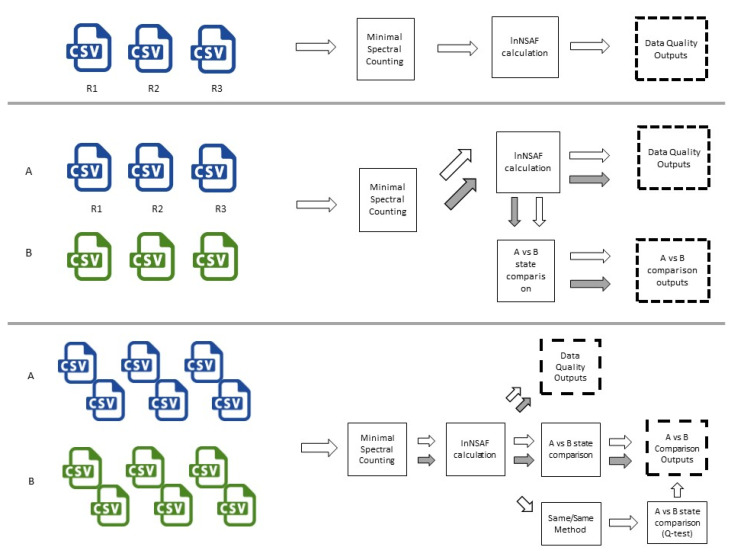
PeptideWitch works by taking in one of three different kind of input combinations. Depending on the combination, a different workflow is executed with different results provided to the end-user. White arrows track the process path for high-stringency data while gray arrows track the parallel execution of the unfiltered ‘All Protein’ process.

A schematic diagram of the processing that occurs, depending on the nature of the inputs, is presented in Figure 1.

#### 2.2.1. Data Quality Analysis

When three replicates of one sample are submitted, data quality analysis is performed, which returns two csv output files per test state. The first contains the highly-stringent protein identification data as determined by the MSC rules applied as described previously, and the second contains a list of all protein identifiers within that particular state, unfiltered, termed ‘All Proteins’. The MSC process generates a collated list of reproducibly identified proteins present in all replicates, which thereby constitutes a high-stringency dataset with random noise filtered out on the basis of being non-reproducible [7]. The ‘All Proteins’ list contains the high-stringency data as well as those proteins which are not reproducibly identified. Protein data are represented with a single row corresponding to a particular protein identifier with the corresponding spectral counts, NSAF Values and scores from each replicate in individual columns.

#### 2.2.2. Control vs. Treatment Comparison Data

When at least three replicates each of two sample states are submitted, PeptideWitch performs a control vs. treatment quantitative analysis of the data using a Students *t*-test for shared protein identifications of both highly stringent and unfiltered data separately. The data streams are split this way into low- and high-stringency results (All Proteins vs. High Stringency) to demonstrate by contrast the effect of minimum spectral counting. Low-stringency data are also presented for the sake of transparency so that users can understand why particular proteins of interest may fail to appear in the high-stringency data, usually because they failed to meet the criteria necessary to be categorised as highly stringent.

Additionally, outputs are sorted into upregulated, downregulated, unchanged, and ‘unique to one state only’ data files, with the latter generated only for the high-stringency data. All comparisons are made relative to the control group, which is user-defined as the first state input to the system. Shared and unique protein identifiers between the input states are displayed in a Venn Diagram, and additional graphical representations are produced as outlined in Results.

#### 2.2.3. Same/Same Analysis Processing and Outputs

If six replicates are provided for each of the two sample states, the replicates of the defined control state are sorted into triplet non-redundant combinations and iteratively compared against each other using a BH modified *t*-test as outlined previously [21]. For each possible combination, the protein-quantitation false discovery rate (PQ-FDR) is calculated—this is the percentage of proteins that appear to be differentially expressed between biologically identical sets of replicates, which are then defined as false discoveries at the protein quantitation level. The test is repeated with the significance cut-off value incrementing over a scale from 0 to 1 in increments of 0.01, and the data are output in graphical form as a plot of BH Q value threshold against PQ-FDR. An average from all ten non-redundant BH tests for each state is produced and those are averaged to produce a single experimentally generated threshold value for the BH test that gives 1% PQ-FDR, which can then be used as the threshold for comparisons between multiple states. PeptideWitch then repeats each of the control vs. treatment comparison *t*-tests using the BH method with the new significance threshold value in place of the standard 0.05 cut-off. Finally, refined versions of upregulated, unchanged, downregulated and combined results are output as new csv files.

A feature table showing the output files produced, depending on the number and type of the inputs, is presented in Table 1. 

## 3. Results

The results output by the software are in the form of csv data spreadsheets and graphical outputs, as described above. These data visualisations are illustrated in the following sections using an example dataset from a previous research study published by our laboratory, involving identification and label-free quantitation of proteins from two cultivars of rice subjected to drought stress, Nipponbare and IAC1131 [11].

The raw data are available from the Pride repository, and PSM algorithm outputs are also available via the PeptideWitch website for use as an illustrative example. Users can download a zipped file containing fifteen csv files of protein identification output data. These consist of six replicates of IAC1131 control, six replicates of Nipponbare control and three replicates of Nipponbare extreme drought stress. When these are uploaded to the PeptideWitch module and processed, outputs are generated, as described in Methods.

### 3.1. Selected Examples of the Graphical Visualisation Data Outputs

#### 3.1.1. Venn Diagrams

**Figure 2:**
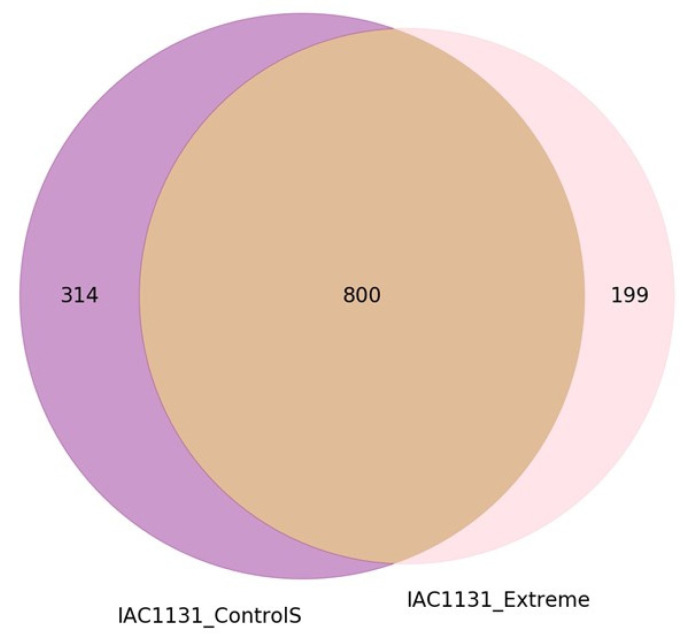
Proportional Venn diagram output by PeptideWitch showing the number of high-stringency proteins reproducibly identified in either sample, or both.

The first question in many comparative proteomics analysis experiments involving two samples is how many proteins are found in either sample state uniquely, and how many are found in both samples irrespective of relative quantitation. One of the simplest and most informative ways to present this information is in the form of a proportional Venn diagram, as shown in Figure 2 for the control and extreme drought stress IAC1131 rice.

#### 3.1.2. Volcano Plots

**Figure 3:**
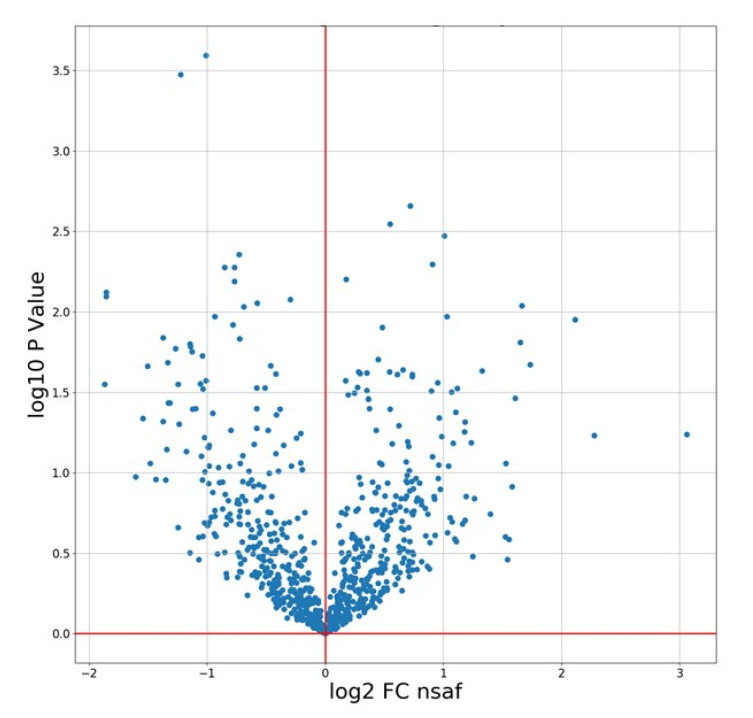
Volcano plot produced by PeptideWitch showing the quantitative comparison between control and extreme drought in IAC 1131 rice data using normalized spectral factors. y-axis represents log10 *p*-value based on Students *t*-test, x-axis represents log2 fold-change.

Volcano plots are a widely used representation for comparing protein identification between two states, showing the fold-change directionally away from a central x-axis, and the statistical significance of the observed fold change along the y-axis. A volcano plot of NSAF data generated from comparison between control and extreme drought stress IAC1131 rice is shown in Figure 3.

#### 3.1.3. Heat Maps

**Figure 4:**
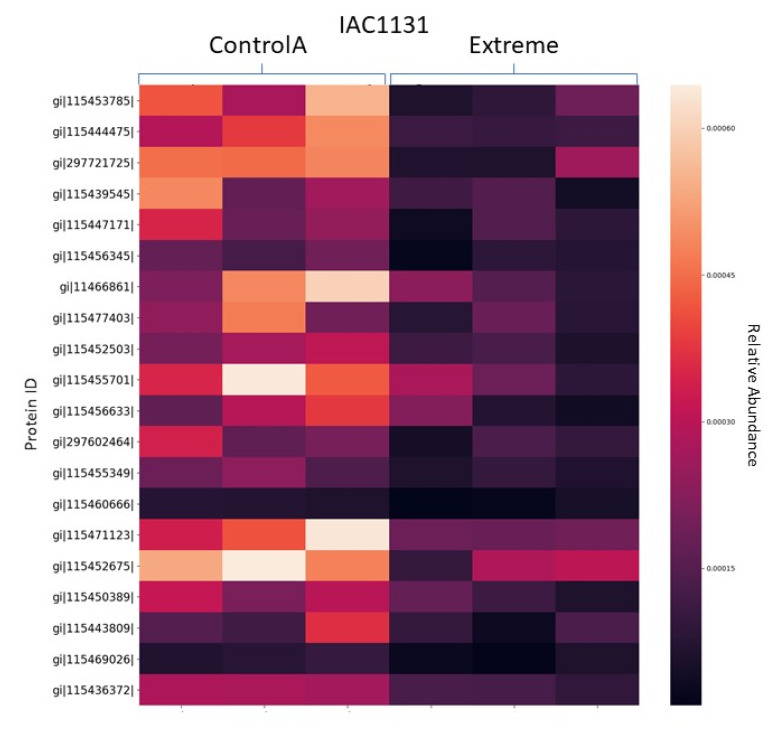
Heatmap produced by PeptideWitch showing the relative intensities of individual replicates for the top 20 most significant differentially expressed proteins in a comparison between control and extreme drought in IAC 1131 rice data.

Heatmaps are used to display relative quantitation values of identified proteins and how these differ across replicates and between samples. A heat map of logNSAF data generated from a comparison between control and extreme drought stress IAC1131 rice, displaying the top 20 most differentially expressed proteins, is shown in Figure 4.

#### 3.1.4. Histograms of *p*-Value Distributions

**Figure 5:**
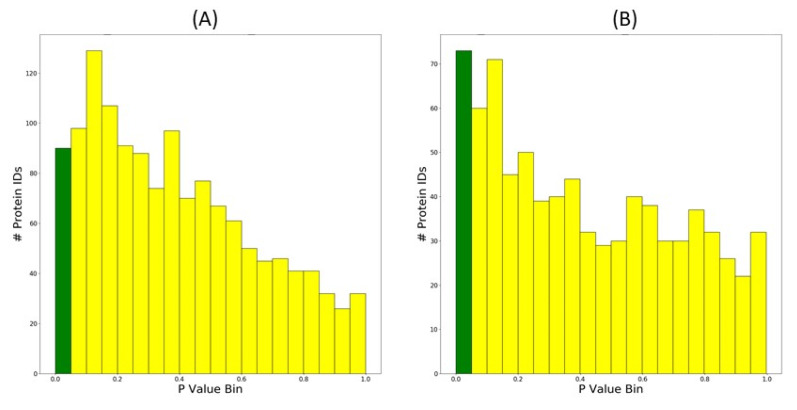
Histograms output by PeptideWitch indicating distribution of *p*-values from a Students *t*-test performed based on label-free quantitation in control and extreme drought from IAC 1131 rice data. (**A**) includes all protein data, while (**B**) includes only high-stringency protein data. The dark green bar represents *p*-values less than 0.05.

Histograms of *p*-value distributions are becoming more widely used in the proteomics field, because they provide a readily available assessment of the effect size within a qualitative proteomics dataset [30]. Histograms of *p*-values generated for high-stringency data, and all protein data, resulting from a comparison between control and extreme drought stress in IAC1131 rice are shown in Figure 5. This demonstrates that in the high-stringency data, the statistically significant differences represent a higher proportion of the overall population, so it is easier to see discern the effect.

#### 3.1.5. Protein Quantitation-False Discovery Rate Plot

**Figure 6:**
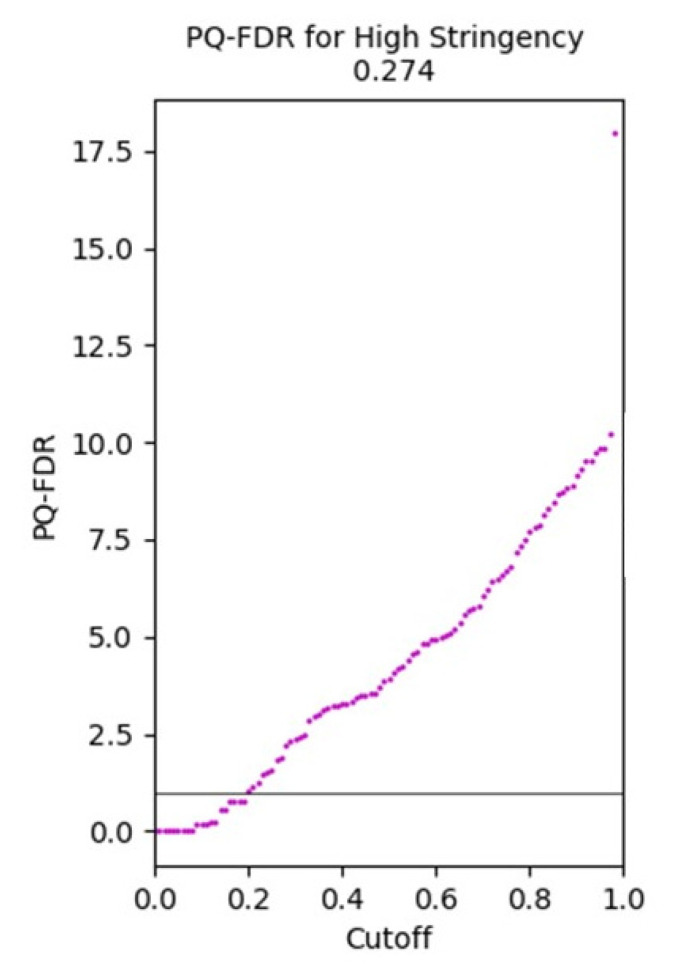
Graphical output produced by PeptideWitch showing a plot of Benjamini-Hochberg adjusted Q value against Protein Quantitation-False Discovery Rate following same-same permutation analysis of six replicates from IAC1131 rice control data.

One additional graphical output conditionally produced by PeptideWitch is a plot showing the relationship between BH Q value threshold and PQ-FDR for a given dataset with six replicates submitted, using an iterative permutation approach to compare sets of triplicates against each other. In the example shown in Figure 6, the results indicate that employing an empirically determined Q value of 0.274 should generate a PQ-FDR of 1% or less, so that criteria can be applied in subsequent analyses between controls and different sample states.

## 4. Discussion

There are numerous options already available in the field of proteomics for data analysis and visualisation, including software such as LFQ-Analyst [31] and PANDA-View [32]. LFQ-Analyst produces highly useful data visualisations including volcano plots and heat maps, and also incorporates pathway enrichment tools, but it is designed to work exclusively with outputs from the MaxQuant PSM search engine. PANDA-View is able to work with outputs from various search engines, and provides multiple data visualization outputs plus extra features such as normalization and missing value imputation, but it is currently available only as source code which requires installation prior to operation. Our intention in the development of PeptideWitch was to combine some of the best features of software such as these, by making the software able to process data from a variety of different PSM search engines, and freely available via a simple web-based interface.

While PeptideWitch incorporates many of the underlying Scrappy processes [7] using newly developed code, the platform adds the following additional functionalities: (1) a proportional Venn diagram showing distribution of proteins in two-sample comparisons, (2) a volcano plot for two-sample comparisons, (3) a heatmap of the top 20 differentially expressed proteins, (4) *p*-value histograms for two-sample comparisons, and (5) Same-Same MTC analysis when six replicates of a designated control sample are provided. The facile generation of a coherent series of high-quality visual outputs is a great improvement and will be of great benefit to researchers in presenting their data.

In addition, the online front-end developed for PeptideWitch allows users anywhere to upload and process their data without having to clone the code repository. That being said, PeptideWitch is open for developmental contributions; the manner in which the code is structured allows for a modular approach in adding or subtracting features so that other users can tweak the platform to suit their analysis requirements.

Another new feature of the PeptideWitch software is that statistical tests between samples are carried out on high-stringency data generated using MSC rules, but also on the low-stringency data in parallel. This is done mainly for transparency reasons, so users can track all of the proteins identified in each of their replicates. Users are encouraged to use the high-stringency datasets for their subsequent research and to treat the low-stringency data as demonstrative.

The software also allows for data to be re-analysed using a same-same permutation analysis to determine experimentally derived BH cut-off values, which can then be applied to a two-sample comparison. We have shown this method to be a more permissive approach to differential protein quantitation whilst correcting for the multiple testing problem, allowing researchers to broaden the scope of potential differentially expressed proteins to be validated by downstream follow-up analysis.

There are several obvious avenues for future improvements to PeptideWitch. The ability to compare high-stringency data with ‘mixed’-stringency datasets would allow for interesting qualitative comparisons. At present, the split between high-stringency and low-stringency data means that some protein identifiers that may conform to one but not both criteria for high-stringency (i.e., minimum spectral count, presence in all replicates) are excluded from being analysed as part of the high-stringency data. Implementing this change may allow for the recovery of more potential differentially expressed proteins, which would also need to be validated by further statistical or experimental means. One other additional change which is currently in development is to incorporate the use of shared or distributed NSAF values, which have been shown previously to provide more precise label free quantitation, especially for lower abundance proteins [33,34].

## 5. Conclusions

PeptideWitch is a data analysis tool for shotgun proteomic analysis designed to output high-stringency data based on minimal spectral counting combination of at least three replicates of a given sample. The program performs statistical analyses and outputs a series of information-rich images which allow the user to easily visualise data quality and differential protein expression between samples.

## Figures and Tables

**Table 1:** Files output by PeptideWitch, depending on the nature of input data files.

Number of States	Two + States Upload		Single State Upload	
Number of Replicates	<6 Replicates	6 Replicates	<6 Replicates	6 Replicates
Upregulated Protein ID csv files	✅	✅		
Downregulated Protein ID csv files	✅	✅		
Unchanged Protein ID csv files	✅	✅		
Unique Protein ID csv files	✅	✅		
Venn Diagrams	✅	✅		
Volcano Plots	✅	✅		
Top 20 Heatmap	✅	✅		
*p*-value Histograms	✅	✅		
Inter-state PCAs of lnNSAF and SpC	✅	✅		
Same-Same combinatorial PCAs		✅		✅
PQ-FDR plot		✅		✅
List of All Protein IDs	✅	✅	✅	✅
List of High Stringency protein IDs	✅	✅	✅	✅

## Abbreviations

AUC	Area Under the Curve
BH	Benjamini-Hochberg
FDR	False Discovery Rate
GPM	Global Proteome Machine
MSC	Minimum Spectral Counting
NSAF	Normalized Spectral Abundance Factors
PQ-FDR	Protein Quantitation-False Discovery Rate
SpC	Spectral Count

## References

[1] Lundgren D.H., Hwang S.-I., Wu L., Han D. (2010). Role of spectral counting in quantitative proteomics. Expert Rev. Proteomics.

[2] Choi M., Eren-Dogu Z.F., Colangelo C., Cottrell J., Hoopmann M.R., Kapp E.A., Kim S., Lam H., Neubert T.A., Palmblad M. (2017). ABRF Proteome Informatics Research Group (iPRG) 2015 study: Detection of differentially abundant proteins in label-free quantitative LC-MS/MS experiments. J. Proteome Res..

[3] Dowle A.A., Wilson J., Thomas J.R. (2016). Comparing the diagnostic classification accuracy of iTRAQ, peak-area, spectral-counting, and emPAI methods for relative quantification in expression proteomics. J. Proteome Res..

[4] Old W.M., Meyer-Arendt K., Aveline-Wolf L., Pierce K.G., Mendoza A., Sevinsky J.R., Resing K.A., Ahn N.G. (2005). Comparison of label-free methods for quantifying human proteins by shotgun proteomics. Mol. Cell. Proteomics.

[5] Paoletti A.C., Parmely T.J., Tomomori-Sato C., Sato S., Zhu D., Conaway R.C., Conaway J.W., Florens L., Washburn M.P. (2006). Quantitative proteomic analysis of distinct mammalian mediator complexes using normalized spectral abundance factors. Proc. Natl. Acad. Sci. USA.

[6] Zybailov B., Mosley A.L., Sardiu M.E., Coleman M.K., Florens L., Washburn M.P. (2006). Statistical analysis of membrane proteome expression changes in *Saccharomyces cerevisiae*. J. Proteome Res..

[7] Neilson K.A., Keighley T., Pascovici D., Cooke B., Haynes P.A., Zhou M., Veenstra T. (2013). Label-Free Quantitative Shotgun Proteomics Using Normalized Spectral Abundance Factors. Proteomics for Biomarker Discovery. Methods in Molecular Biology (Methods and Protocols).

[8] Neilson K.A., Ali N.A., Muralidharan S., Mirzaei M., Mariani M., Assadourian G., Lee A., Van Sluyter S.C., Haynes P.A. (2011). Less label, more free: Approaches in label-free quantitative mass spectrometry. Proteomics.

[9] George I.S., Fennell A.Y., Haynes P.A. (2018). Shotgun proteomic analysis of photoperiod regulated dormancy induction in grapevine. J. Proteomics.

[10] George I.S., Fennell A.Y., Haynes P.A. (2015). Protein identification and quantification from riverbank grape, *Vitis riparia*: Comparing SDS-PAGE and FASP-GPF techniques for shotgun proteomic analysis. Proteomics.

[11] Wu Y., Mirzaei M., Pascovici D., Chick J.M., Atwell B.J., Haynes P.A. (2016). Quantitative proteomic analysis of two different rice varieties reveals that drought tolerance is correlated with reduced abundance of photosynthetic machinery and increased abundance of ClpD1 protease. J. Proteomics.

[12] Vaibhav V., Thompson E.L., Raftos D.A., Haynes P.A. (2018). Potential protein biomarkers of QX disease resistance in selectively bred Sydney Rock Oysters. Aquaculture.

[13] Muralidharan S., Thompson E., Raftos D., Birch G., Haynes P.A. (2012). Quantitative proteomics of heavy metal stress responses in Sydney rock oysters. J. Proteomics.

[14] Mirzaei M., Pascovici D., Atwell B.J., Haynes P.A. (2012). Differential regulation of aquaporins, small GTP ases and V-ATP ases proteins in rice leaves subjected to drought stress and recovery. Proteomics.

[15] Emery S.J., Lacey E., Haynes P.A. (2015). Quantitative proteomic analysis of *Giardia duodenalis* Assemblage A: A baseline for host, assemblage, and isolate variation. Proteomics.

[16] Emery S.J., Lacey E., Haynes P.A. (2015). Data from a proteomic baseline study of Assemblage A in Giardia duodenalis. Data Brief.

[17] Emery S.J., Pascovi D., Lacey E., Haynes P.A. (2015). The generation gap: Proteome changes and strain variation during encystation in *Giardia duodenalis*. Mol. Biochem. Parasitol..

[18] Emery S.J., van Sluyter S., Haynes P.A. (2014). Proteomic analysis in *Giardia duodenalis* yields insights into strain virulence and antigenic variation. Proteomics.

[19] Taleahmad S., Mirzaei M., Parker L.M., Hassani S.-N., Mollamohammadi S., Sharifi-Zarchi A., Haynes P.A., Baharvand H., Salekdeh G.H. (2015). Proteome analysis of ground state pluripotency. Sci. Rep..

[20] Francis H.M., Mirzaei M., Pardey M.C., Haynes P.A., Cornish J.L. (2013). Proteomic analysis of the dorsal and ventral hippocampus of rats maintained on a high fat and refined sugar diet. Proteomics.

[21] Franklin J.L., Mirzaei M., Wearne T.A., Sauer M.K., Homewood J., Goodchild A.K., Haynes P.A., Cornish J.L. (2016). Quantitative shotgun proteomics reveals extensive changes to the proteome of the orbitofrontal cortex in rats that are hyperactive following withdrawal from a high sugar diet. Proteomics.

[22] Handler D.C.L., Haynes P.A. (2019). An experimentally-derived measure of inter-replicate variation in reference samples: The same-same permutation methodology. bioRxiv..

[23] Bender R., Lange S. (2001). Adjusting for multiple testing–when and how?. J. Clin. Epidemiol..

[24] Benjamini Y., Hochberg Y. (1995). Controlling the false discovery rate: A practical and powerful approach to multiple testing. J. R. Stat. Soc. Series B.

[25] Storey J.D., Tibshirani R. (2003). Statistical significance for genomewide studies. Proc. Natl. Acad. Sci. USA.

[26] Bitbucket. www.bitbucket.com/peptidewitch/peptidewitch.

[27] Craig R., Beavis R.C. (2004). TANDEM: Matching proteins with tandem mass spectra. Bioinformatics.

[28] Solntsev S.K., Shortreed M.R., Frey B.L., Smith L.M. (2018). Enhanced global post-translational modification discovery with MetaMorpheus. J. Proteome Res..

[29] Locard-Paulet M., Bouyssie D., Froment C., Burlet-Schiltz O., Jensen L.J. (2020). Comparing 22 popular phosphor proteomics pipelines for peptide identification and site localization. J. Proteome Res..

[30] Pascovici D., Handler D.C., Wu J.X., Haynes P.A. (2016). Multiple testing corrections in quantitative proteomics: A useful but blunt tool. Proteomics.

[31] Shah A.D., Goode R.J.A., Huang C., Powell D.R., Schittenhelm R.B. (2020). LFQ-analyst: An Easy-to-use interactive web platform to analyze and visualize label-free proteomics data preprocessed with MaxQuant. J. Proteome Res..

[32] Chang C., Xu K., Guo C., Wang J., Yan Q., Zhang J., He F., Zhu Y. (2018). PANDA-view: An easy-to-use tool for statistical analysis and visualization of quantitative proteomics data. Bioinformatics.

[33] Lee H.Y., Kim E.G., Jung H.R., Jung J.W., Kim H.B., Cho J.W., Kim K.M., Yi E.C. (2019). Refinements of LC-MS/MS spectral counting statistics improve quantification of low abundance proteins. Sci. Rep..

[34] Zhang Y., Wen Z., Washburn M.P., Florens L. (2015). Improving label-free quantitative proteomics strategies by distributing shared peptides and stabilizing variance. Anal. Chem..

